# Tanshinone IIA Restrains Hepatocellular Carcinoma Progression by Regulating METTL3-Mediated m6A Modification of TRIB3 mRNA

**DOI:** 10.5152/tjg.2025.24304

**Published:** 2025-02-04

**Authors:** Ying Jiang, Xinjie Wang, Zhenyang Wang, Shengliang Zhang, Jianhua Wang, Xianglong Wang, Yang Zhang

**Affiliations:** Department of Hepatobiliary Surgery, The First Affiliated Hospital of Guizhou University of Chinese Medicine, Guiyang City, Guizhou Province, China

**Keywords:** Hepatocellular carcinoma,, tanshinone IIA,, N6-methyladenosine,, TRIB3

## Abstract

**Background/Aims:**

Hepatocellular carcinoma (HCC) is a molecularly heterogeneous solid malignancy that carries a dismal prognosis. Tanshinone IIA (Tan-IIA) is involved in the regulation of N6-methyladenosine (m6A) modification and plays an anti-tumor role in HCC, but whether Tan-IIA regulates HCC by mediating m6A modification is unclear.

**Methods and Materials:**

Cell apoptosis, invasion, proliferation, viability, and stemness were estimated with flow cytometry, transwell, 5-ethynyl-2’-deoxyuridine, 3-[4,5-dimethylthiazol-2-yl]-2,5-diphenyl tetrazolium bromide, and sphere-forming assays. Methyltransferase-like 14 (METTL14) and 3 (METTL3) mRNA and protein levels were detected with reverse transcription-quantitative polymerase chain reaction and western blotting. Total m6A levels were measured using an m6A RNA methylation quantification kit. A possible regulation of tribbles pseudokinase-3 (TRIB3) expression by METTL3 in an m6A-modified manner was predicted through RM2Target and SRAMP and verified by m6A methylated RNA immunoprecipitation (MeRIP) and RIP. Mouse xenograft models assessed the action of Tan-IIA in HCC tumorigenesis.

**Results:**

Tanshinone IIA restrained HCC cell viability, proliferation, invasion, and stemness, and induced HCC cell apoptosis *invitro,* as well as repressed tumor growth in xenograft models. METTL3 and TRIB3 were upregulated in HCC samples and downregulated in Tan-IIA-treated HCC cells and xenograft tumors. METTL3 facilitated HCC cell viability, proliferation, invasion, and stemness by enhancing TRIB3 mRNA stability through m6A modification. Tan-IIA played its role by downregulating TRIB3, and Tan-IIA mediated TRIB3 expression by METTL3.

**Conclusion:**

Tanshinone IIA restrained HCC progression by regulating METTL3-mediated m6A modification of TRIB3 mRNA, offering evidence to support the clinical translation of Tan-IIA.

**Main Points**

- Tan-IIA represses HCC cell proliferation, invasion, and stemness.
- METTL3 mediates m6A modification of TRIB3 mRNA.
- Tan-IIA works by regulating METTL3-mediated m6A modification of TRIB3 mRNA.

## Introduction

Hepatocellular carcinoma (HCC) is an aggressive primary liver tumor that occurs commonly in cirrhosis or chronic liver inflammation.^1^ It is insidious with a slow progression, and most patients are often diagnosed at a later stage, which makes treatment more challenging.^2^ Currently, patients with intermediate and advanced HCC are mainly treated with transarterial chemoembolization, immunotherapy, and chemotherapy, but the final therapeutic effect is still very limited, with a high recurrence rate.^3,4^ Therefore, developing new therapeutic agents for HCC is essential to improve HCC.^5^

Tanshinone IIA (Tan-IIA), an active lipophilic component of Salvia miltiorrhiza extract, has various pharmacological activities, such as anti-atherosclerosis, cardio-protection, neuro-protection, and anti-tumor.^6,7^ Available evidence indicates that Tan-IIA exerts anti-tumor efficacy by inhibiting tumor cell proliferation, inhibiting tumor angiogenesis, and inducing tumor cell differentiation and apoptosis.^8^ Mechanistically, Tan-IIA regulates the expression of tumor promoters or inhibitors in HCC.^8^ For instance, Tan-IIA can inhibit liver cancer growth by mediating the SMAD7/YAP pathway.^9^ In addition, Tan-IIA remodels the Rho GTPase-mediated actin cytoskeleton, resulting in the regulation of cancer cell morphology and motility.^10^ Although the effects of Tan-IIA on HCC have been identified, there are fewer studies in which Tan-IIA works in an epigenetic-dependent manner.

As the most abundant epitranscriptomic modification in eukaryotic RNAs, N6-methyladenosine (m6A) participates in tumorigenesis, progression, metabolism, and metastasis.^11,12^ Modification of m6A is a dynamic process of methylation and demethylation, and methyltransferases and demethylases maintain the balance of this process, which can cause different degrees of damage to the organism when this balance is broken.^13,14^ A substantial number of papers have been published to support that m6A regulators are biomarkers of HCC.^15^ Abnormal alterations in m6A levels and the m6A methylase complex affect HCC progression by either inhibiting or promoting the expression of HCC-associated genes.^16,17^ Methyltransferase-like 14 (METTL14) and 3 (METTL3) have been reported to participate in HCC progression.^18,19^ Notably, Tan-IIA has been reported to attenuate cardiac hypertrophy by modulating m6A modifications,^20^ but whether Tan-IIA can play a role in HCC by mediating METTL3 and METTL14 expression levels is unclear.

This study focused on investigating whether Tan-IIA can inhibit HCC progression by modulating METTL3- and METTL14-dependent M6A modifications, which might contribute to exploiting more effective therapeutic strategies for HCC.

## Materials and Methods

### Cell Culture and Tan-IIA Treatment

The growth media for the HCC cell lines Hep3B (cat#CL-0102, Procell, Wuhan, China) and Huh-7 (cat#CL-0120, Procell) were minimum essential medium (MEM) containing NEAA (Procell) or Dulbecco’s MEM (Procell) (cat#PM150210) supplemented with 10% fetal bovine serum (FBS) (Gibco™, Thermo, Waltham, MA, USA) and 1% P/S solution (Procell), respectively. Human hepatic immortalized cells THLE-2 (cat#CL-0833, Procell) were cultured in their specialized medium (Procell). Tan-IIA treatment was performed by culturing THLE-2 and HCC cell lines in a medium containing Tan-IIA (0, 10, 20, and/or 40 µM) (Aladdin, Shanghai, China) for 48 h.

### Plasmids and Small Interfering (si) RNAs

All siRNAs were chemically synthesized by GeneCreate (Wuhan, China), including negative control (si-NC), si-METTL3#1, si-METTL3#2, and si-METTL3#3 In the METTL3 interference assay, three siRNAs of this gene were merged into a pool of siRNAs to co-transfect HCC cell lines to avoid off-target effects. Recombinant plasmids pcDNA-TRIB3 (TRIB3) and pcDNA-METTL3 (METTL3) were generated by GeneCreate. Transfection of plasmids or siRNA into HCC cells was accomplished using the jetPRIME Transfection Reagent (Polyplus, New York, NY, USA) according to the manufacturer’s guidelines. For double transfection, oligonucleotides (50 nM) and overexpression plasmids (2 μg) were mixed, followed by co-incubation with the jetPRIME reagent (5 μL).

### [4,5-Dimethylthiazol-2-yl]-2,5 Diphenyl Tetrazolium Bromide (MTT) Assay

About 5 × 103 THLE-2 or HCC cells were seeded in 96-well plates and treated with or without Tan-IIA for 48 h. Determination of cell viability was made with the MTT assay kit (Beyotime, Shanghai, China). Ten microliters of MTT solution (5 mg/mL) was added and incubated for 4 h, followed by dissolution of precipitated crystals with 100 μL of formazan solution. Absorbance was monitored using a Multiskan FC microplate reader (Thermo) at 570 nm.

### 5-Ethynyl-2’-Deoxyuridine (EdU) Assay

Hepatocellular carcinoma cells (5 × 103) in different groups were incubated for 48 h, followed by incubation with the EdU working solution (10 µM) (Beyotime) for 2 h. For fixation and improvement of cell membrane permeability, cells were incubated with 4% paraformaldehyde (Beyotime) and 0.5% Triton X-100 (Beyotime) for 15 min, respectively. EdU-positive cells were observed using a fluorescence microscope (Leica, Germany) after labeling the nuclei with DAPI (Beyotime), followed by analysis with ImageJ v 1.51 software (NIH, Bethesda, MD, USA).

### Flow Cytometry Assay

HCC cells (5 × 103) were harvested after incubation for 48 h. Assessment of cell apoptosis was conducted with the annexin V-FITC/PI apoptosis kit (Multi Sciences, Hangzhou, China) following the operating instructions packed with the kit. Early and late apoptotic cells were evaluated by flow cytometry (BD FACSCanto II; BD Biosciences, Franklin Lakes, USA).

### Transwell Invasion Assay

Cell invasive ability was estimated with the Transwell chamber system (Corning, New York, USA). In brief, approximately 1 × 103 HCC cells were seeded into the upper chamber pre-coated with Matrigel (Corning). Six hundred microliters of culture media with 10% FBS were added outside the chambers. Twenty-four hours later, the cells were stained with 0.1% crystal violet (Beyotime). Imaging and counting of invasive cells were undertaken using an inverted microscope system (IX73, Olympus, Japan).

### Sphere-Forming Assay

Cell stemness was analyzed by sphere-forming assays. Subsequently, single cells (1 × 103) were plated in Ultra Low Attachment plates (Corning) in serum-free medium supplemented with epidermal growth factor (20 ng/mL) (Sigma), basic fibroblast growth factor (20 ng/mL) (Sigma), 1% B27 (iCell), and 1% methyl cellulose (Cytiva, Shanghai, China) for 10 days. Spheroid formation was photographed and assessed using an inverted light microscope (Olympus).

### Database and Bioinformatics Analysis

The TCGA-liver hepatocellular carcinoma (LIHC) dataset used in the study was downloaded from the TCGA data portal (http://gdc-portal.nci.nih.gov/). The potential m6A sites in TRIB3 were predicted by the SRAMP the website (https://www.cuilab.cn/sramp/). The binding relationship between METTL3 and TRIB3 was predicted by the RM2Target website (http://rm2target.canceromics.org/).

### Total RNA Extraction and m6A Quantification

TRIeasy Total RNA Extraction Reagent (Yeasen, Shanghai, China) was used for the isolation of total RNA from HCC cells. Quantification was done by reading the absorbance values at 260 and 280 nm. Changes in overall m6A levels in total RNA (200 ng) were measured using the m6A RNA Methylation Quantification Kit (Abcam, Cambridge, MA, USA) as per the operating directions provided in the kit. Measurement of absorbance was performed with a Multiskan FC microplate reader (Thermo) at 450 nm, followed by analysis of m6A levels according to a standard curve.

### Western Blotting

Tissue samples and cultured cells were homogenized separately in ice-cold RIPA lysing buffer for 30 min. After centrifugation, the supernatants were collected, and protein concentration was measured. Protein samples (30 μg) were separated on sodium dodecyl sulfate polyacrylamide gel electrophoresis and then transferred to a polyvinylidene difluoride membrane. Following incubation with 5% BSA, the membranes were incubated with primary antibodies against METTL3 (1:1000 dilution, Abcam, ab195352), TRIB3 (1:5000 dilution, Abcam, ab75846), and GAPDH (1:10000 dilution, Abcam, ab181602). After incubation with a secondary antibody (1:3000 dilution, Abcam, ab205718), protein signals were developed with a chemiluminescence detection system (Beyotime) and quantified by ImageJ software (NIH).

### Reverse Transcription-Quantitative Polymerase Chain Reaction (RT-qPCR)

Reverse transcription reactions were carried out using the HiScript II 1st Strand cDNA Synthesis Kit (+gDNA wiper) (Vazyme, Nanjing, China). qPCR was undertaken with the AceQ Universal SYBR qPCR Master Mix (Vazyme). Relative mRNA expression levels were determined using the 2^−∆∆CT^ method. The primers synthesized by GeneCreate are displayed in Table 1.

### Study Subjects

All clinical specimens were from the First Affiliated Hospital of Guizhou University of Chinese Medicine, including 33 HCC samples and corresponding para-cancerous tissues. Prior to obtaining the clinical specimens, physicians provided all the necessary information to the patients and their families. Signed informed consent was obtained from the patients. Additionally, none of the enrolled patients had received radiotherapy or chemotherapy. This study conformed to the Declaration of Helsinki and had been approved by the Ethics Committee of the First Affiliated Hospital of Guizhou University of Chinese Medicine (approval number: 20220423S, date: April 13, 2022).

### RNA-Binding Protein Immunoprecipitation (RIP)

Hepatocellular carcinoma cells transfected with si-NC or si-METTL3 were lysed in IP lysis buffer, followed by incubation with protein A/G beads pre-coated with anti-METTL3. After purification, the enrichment of TRIB3 in immunoprecipitated RNAs was analyzed by RT-qPCR.

### m6A-RNA Immunoprecipitation (Me-RIP) Assay

Total RNA (5 μg) from HCC cells was incubated with an m6A-specific antibody (1:500 dilution, Abcam, ab151230) or IgG (1:100 dilution, Abcam, ab109489,), followed by incubation with the protein A/G magnetic beads for 1 h. The m6A level of TRIB3 in immunoprecipitated RNA was analyzed using RT-qPCR.

### Detection of mRNA Stability

For the measurement of RNA stability, transfected HCC cells were processed with actinomycin D for 0, 3, 6, and 9 h. Total RNA was then isolated, and TRIB3 mRNA levels were analyzed via RT-qPCR.

### Animal Experiments

Male BALB/c nude mice (5 weeks old) (Vital River Laboratory, Beijing, China) were used for animal experiments. Huh-7 cells (5 × 106) were injected into the right flank of nude mice (n = 5). The treatment group was administered Tan IIA (10 mg/Kg/d) one week after injection, and an equal amount of DMSO was used as a control. Tumor size was measured using sliding calipers [volume = (length × width 2)/2]. Twenty-two days later, the subcutaneous tumors were excised, fixed in 4% paraformaldehyde, and paraffin-embedded. The paraffin-embedded subcutaneous tumors were cut into 5 μm sections. All tumor sections were used to detect PCNA (1:100 dilution, Abcam, ab92552), METTL3 (1:500 dilution, Abcam, ab195352), and TRIB3 (1:100 dilution, Sangon Biotech, Shanghai, China, D223415) by immunohistochemistry (IHC) staining. Experimentation on animals was performed after approval by the First Affiliated Hospital of Guizhou University of Chinese Medicine Ethics Committee. (approval number: 20220605D, date: June 5, 2022).

### Statistical Analysis

Data were processed with GraphPad Prism 7.0 (GraphPad, La Jolla, CA, USA) and expressed as mean ± standard deviation. Comparisons between the two groups were undertaken using paired and unpaired t-tests. Comparisons of three or more groups were made by one-way ANOVA with Bonferroni post hoc tests. Statistical significance was expressed as *P* < .05.

## Results

### Tan-IIA Restrained HCC Cell Proliferation, Invasion, and Stemness

**Figure 1.**
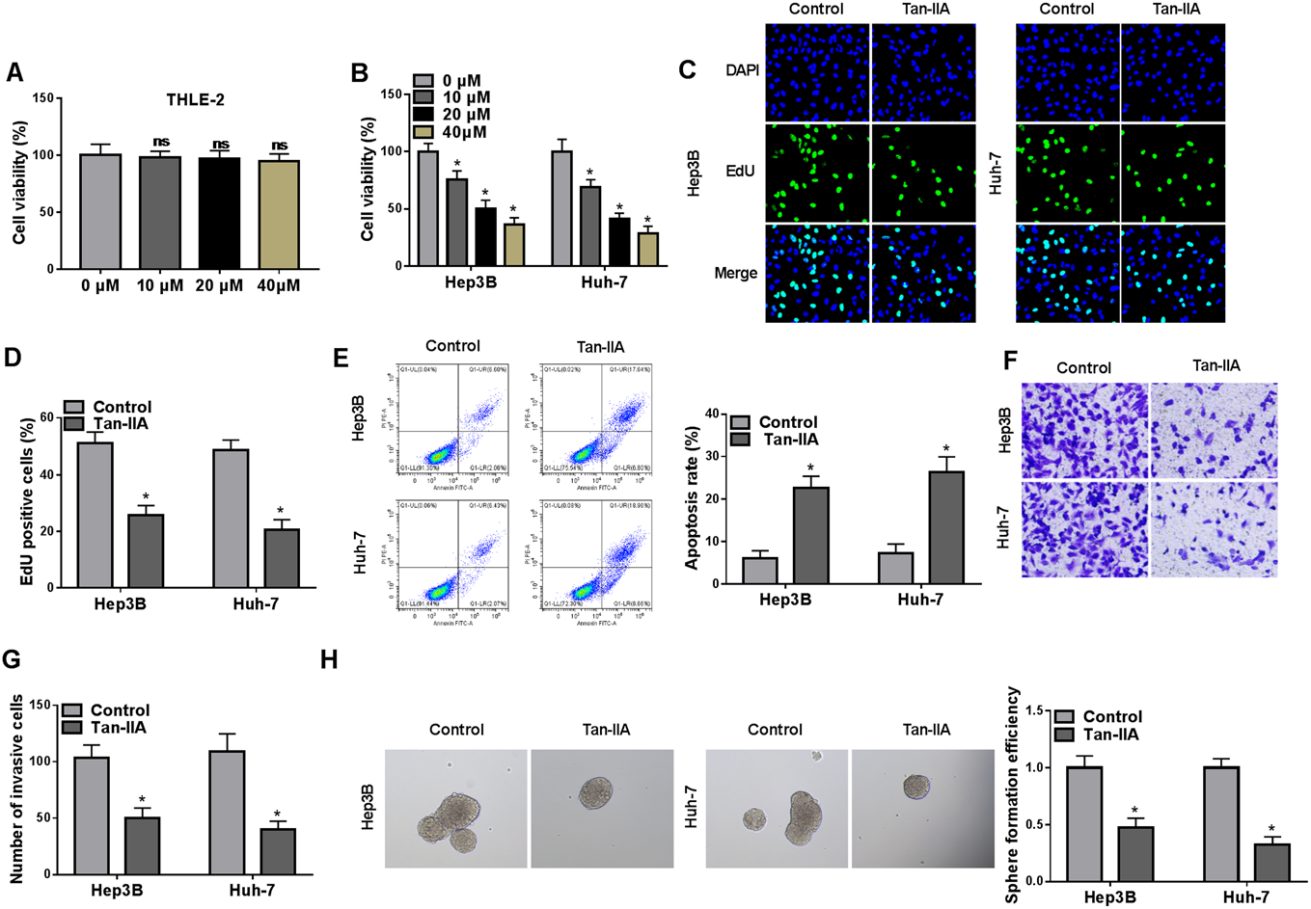
Tan-IIA suppressed HCC cell proliferation, invasion, and stemness. (A and B) MTT assays determined the viability of THLE-2 and HCC cells treated with different concentrations of Tan-IIA (0, 10, 20, and 40 µM). ns: no significant; *P < .05 versus 0 µM. Significant differences were analyzed using one-way ANOVA. (C-H) Effects of Tan-IIA on HCC cell proliferation, apoptosis, invasion, and stemness were estimated with EdU, flow cytometry, transwell invasion, and sphere-forming assays. *P < .05 versus control.

The cytotoxicity of Tan-IIA on THLE-2 cells was first assessed. We observed that Tan-IIA had no effect on cell viability in THLE-2 cells (Figure 1A). Next, we treated HCC cells with different concentrations of Tan-IIA. As expected, Tan-IIA restrained HCC cell viability in a concentration-dependent manner (Figure 1B). The half-inhibitory concentration of Tan-IIA for HCC cells was about 20 µM, so this concentration was used for subsequent analyses. Furthermore, Tan-IIA reduced the proliferative capacity of HCC cells significantly (Figures 1C and D). On the contrary, Tan-IIA had a promoting effect on apoptosis in HCC cells (Figure 1E). In addition, Tan-IIA reduced the invasive and sphere-forming abilities of HCC cells, as evidenced by transwell invasion and sphere-forming assays (Figures 1F and H). Collectively, these results suggested that Tan-IIA played a repressive role in HCC cell proliferation, invasion, and stemness of HCC cells.

### Tan-IIA Inhibited m6A Methylation and METTL3 Expression in HCC Cells

**Figure 2.**
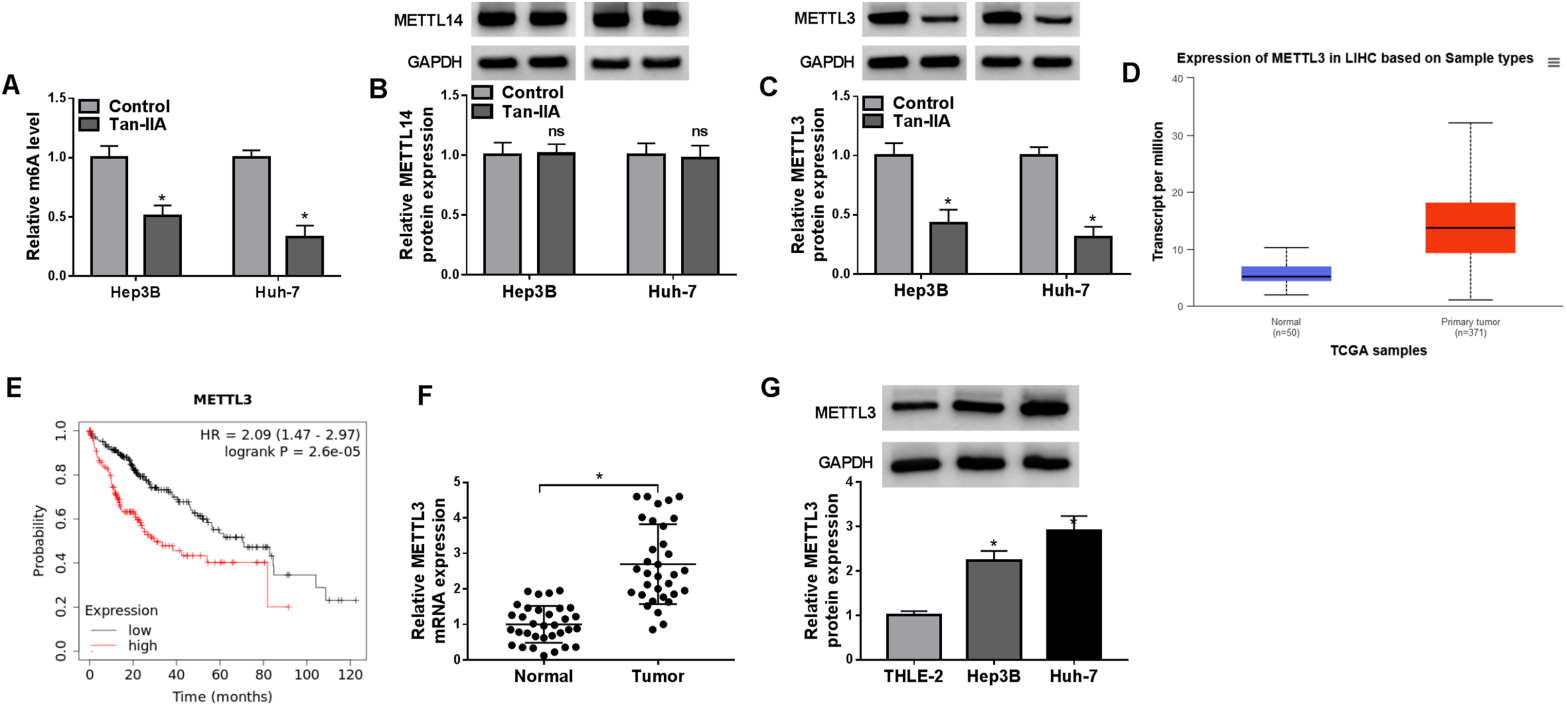
Tan-IIA decreased m6A methylation and suppressed METTL3 expression in HCC cells. (A) Total m6A content was analyzed in Tan-IIA-treated HCC cells. *P < .05 versus control. (B and C) RT-qPCR determined the mRNA levels of METTL14 and METTL3 in Tan-IIA-treated HCC cells. *P < .05 versus control. (D) The expression of METTL3 in TCGA-LIHC dataset. (E) Overall survival of LIHC patients with high or low METTL3 expression in TCGA. (F) RT-qPCR detected METTL3 mRNA levels in HCC samples (n = 33). *P < .05 versus normal. (G) Western blotting evaluated METTL3 protein levels in HCC cells. *P < .05 versus THLE-2.

Given that Tan-IIA modulates m6A RNA modification to alleviate cardiac hypertrophy, we sought to figure out whether Tan-IIA could inhibit malignancy for HCC cells by modulating m6A RNA modification.^20^ We first evaluated the effect of Tan-IIA on the m6A content of total mRNA in HCC cells. The outcomes showed that Tan-IIA resulted in a significant reduction in the abundance of m6A mRNA in HCC cells, suggesting that Tan-IIA may regulate HCC by mediating m6A modification of mRNA (Figure 2A). Because METTL3 and METTL14 are involved in HCC progression,^21,22^ we analyzed whether Tan-IIA mediates m6A modification of mRNAs by regulating METTL3 and METTL14 levels. As displayed in Figures 2B and C, Tan-IIA had no effect on methyltransferase METTL14 protein levels but it inhibited METTL3 protein levels. Analysis of the TCGA-liver hepatocellular carcinoma (LIHC) dataset revealed that METTL3 was upregulated in HCC patients with primary tumors compared with normal tissues, and higher METTL3 levels were linked to poorer overall survival in HCC patients (Figures 2D and E). We then validated the upregulation of METTL3 mRNA levels in 33 HCC samples (Figure 2F). In addition, METTL3 protein levels were markedly elevated in HCC cells (Figure 2G). These data manifested that Tan-IIA might regulate HCC by mediating METTL3-dependent m6A methylation.

### METTL3-Regulated m6A Modification of TRIB3 mRNA

**Figure 3.**
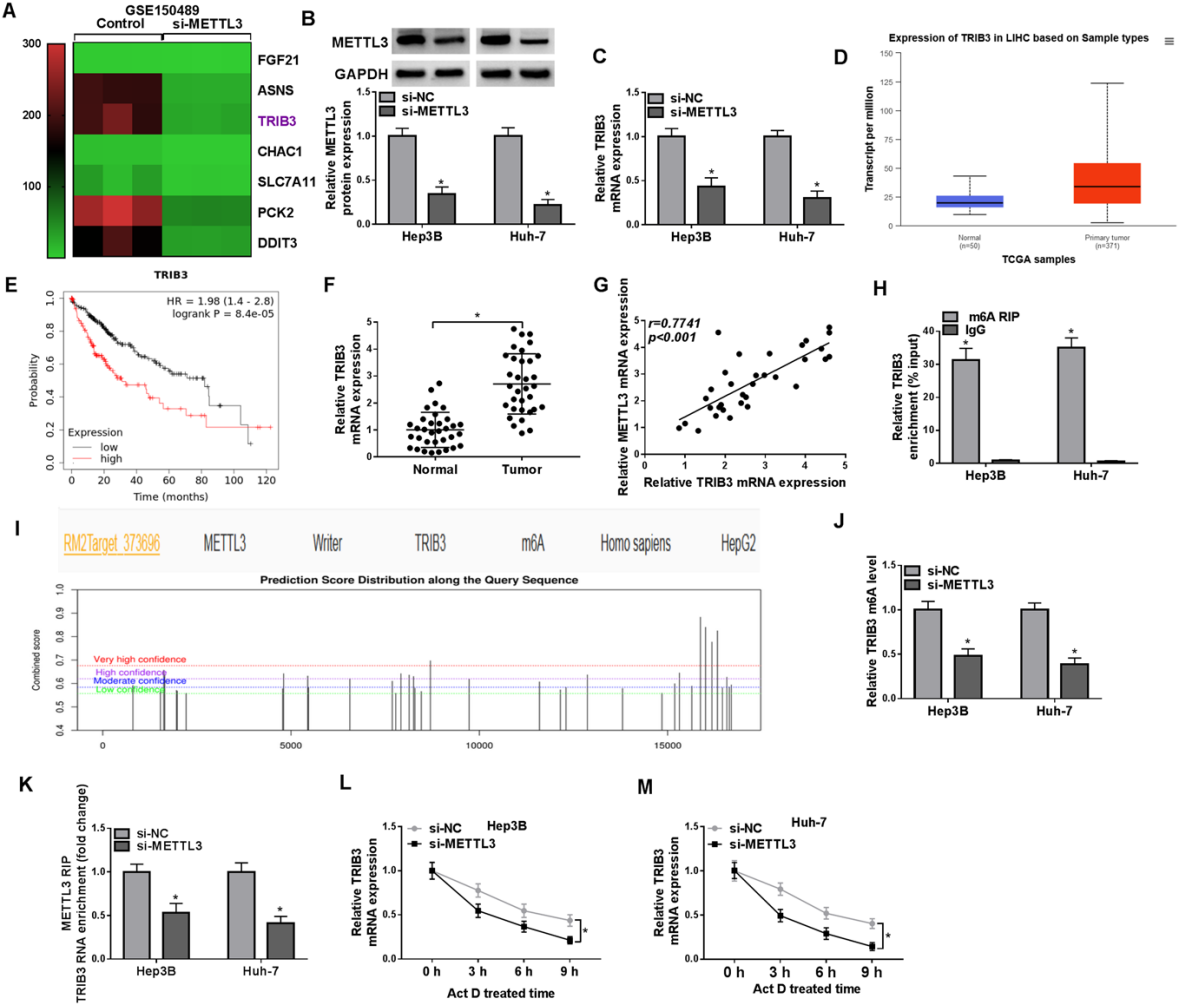
The m6A modification of TRIB3 mRNA was regulated by METTL3. (A) A heatmap showing genes downregulated in Huh7 cells with knockdown of METTL3 from the GEO database (GSE150489). (B and C) Detection of METTL3 protein levels and TRIB3 mRNA levels in si-METTL3-transfected HCC cells. *P < .05 versus si-NC. (D) The expression of TRIB3 in the TCGA-LIHC dataset. (E) Overall survival of LIHC patients with high or low TRIB3 expression in TCGA. (F) Relative mRNA levels of TRIB3 in HCC samples were measured. *P < .05 versus normal. (G) Pearson’s correlation analysis identified a positive correlation between TRIB3 and METTL3 mRNA levels in HCC samples. (H) The m6A modification of TRIB3 mRNA in HCC cells was determined by Me-RIP. *P < .05 versus IgG. (I) The combination of SRAMP and RM2Target predicted that METTL3 may regulate TRIB3 expression through m6A modification. (J) TRIB3 m6A levels in HCC cells transfected with si-NC or si-METTL3 were analyzed by Me-RIP. *P < .05 versus si-NC. (K) The enrichment of TRIB3 mRNA in HCC cells with knockdown of METTL3 was estimated by RIP. *P < .05 versus si-NC. (L and M) TRIB3 mRNA levels were detected by RT-qPCR in METTL3-knockdown HCC cells treated with actinomycin D at the indicated time points. *P < .05 versus si-NC.

To elucidate the molecular mechanisms mediated by METTL3, we searched for downregulated genes upon knockdown of METTL3 using the GEO database GSE150489. As exhibited in Figure 3A, METTL3 silencing significantly decreased TRIB3 expression in Huh7 cells. Subsequently, we interfered with METTL3 in HCC cells (Figure 3B). Regarding TRIB3, its mRNA levels were suppressed in METTL3-knockdown HCC cells (Figure 3C). TCGA-LIHC dataset showed that TRIB3 was upregulated in HCC patients with primary tumors versus normal tissues, and there was an association between higher TRIB3 levels and poorer overall survival in HCC patients (Figures 3D and E). RT-qPCR showed higher levels of TRIB3 mRNA in 33 HCC samples than in normal samples, and it had a positive correlation with METTL3 mRNA levels (Figures 3F and G). Methylated RIP analysis showed that the m6A modification of TRIB3 mRNA was higher compared to the control group (Figure 3H), and there are many methylation modification sites for TRIB3, as predicted by the SRAMP website (Figure 3I). Furthermore, METTL3 knockdown decreased the m6A modification of TRIB3 mRNA in HCC cells (Figure 3J). RNA-binding protein immunoprecipitation assays further revealed that the enrichment of TRIB3 mRNA was significantly reduced following METTL3 silencing (Figure 3K). In addition, silencing METTL3 repressed TRIB3 mRNA levels and its stability (Figures 3L and M). Collectively, these results manifested that METTL3 regulated TRIB3 expression through m6A modification.

### METTL3 Regulated HCC Cell Proliferation, Invasion, and Stemness by TRIB3

**Figure 4.**
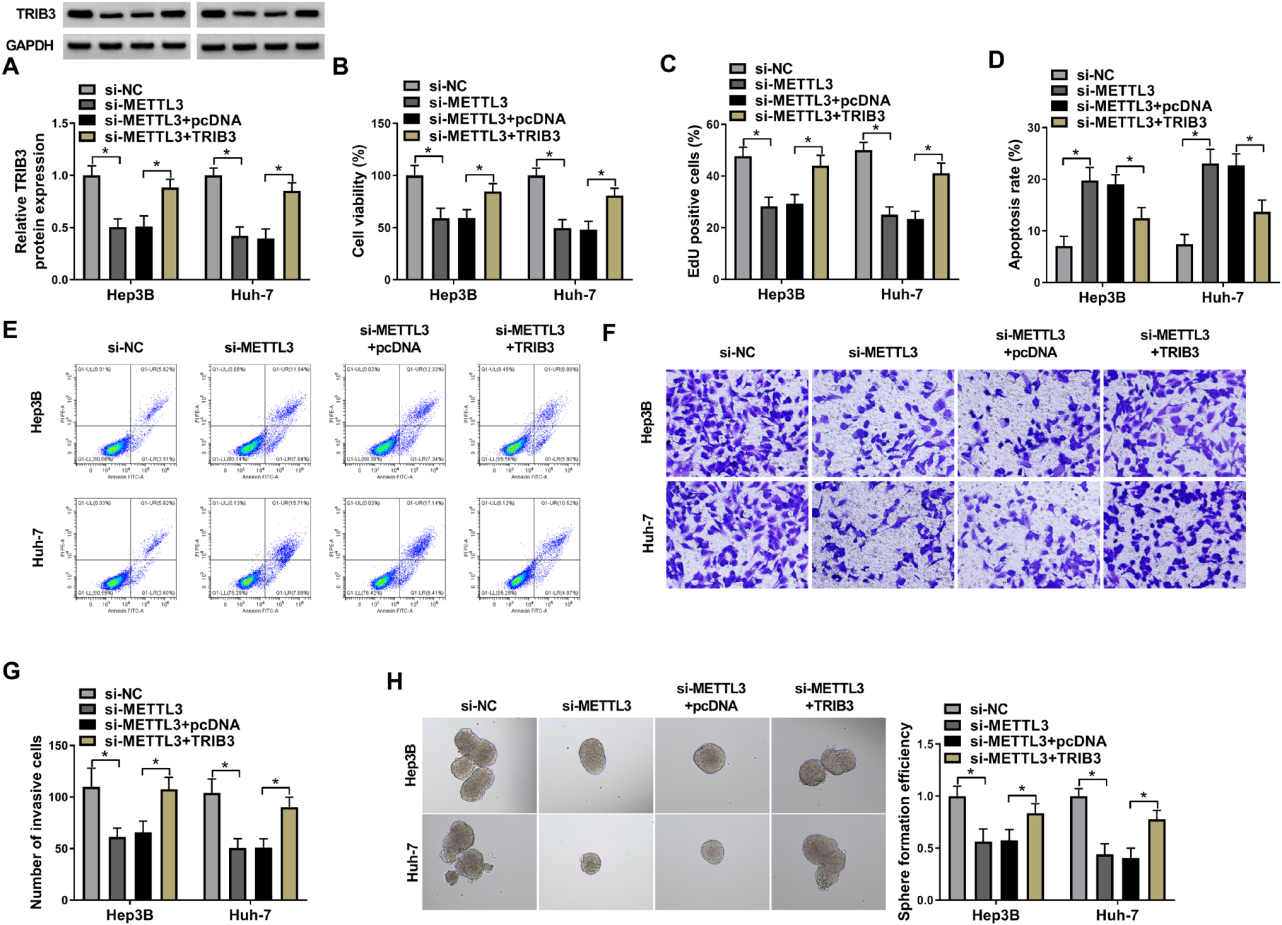
METTL3 regulated TRIB3 expression to mediate HCC cell proliferation, invasion, and stemness. (A) Relative protein levels of TRIB3 in HCC cells with knockdown of METTL3 and in HCC cells with knockdown of METTL3 and overexpression of TRIB3. (B-H) The viability, proliferation, apoptosis, invasion, and stemness of HCC cells with different treatments. *P < .05 versus si-METTL3 or si-METTL3+pcDNA.

To further figure out whether METTL3 mediates HCC progression by TRIB3, TRIB3 overexpression plasmids were introduced into METTL3-knockdown HCC cells. The suppressive influence of METTL3 silencing on TRIB3 protein levels was reversed after TRIB3 overexpression (Figure 4A). Furthermore, METTL3 knockdown decreased cell viability (Figure 4B), restrained cell proliferation (Figure 4C), urged cell apoptosis (Figures 4D and E), as well as repressed cell invasion (Figures 4F and G) and stemness (Figure 4H) in HCC cells, but METTL3 downregulation-mediated these effects were impaired following TRIB3 overexpression. Together, these results suggested that METTL3 restrained HCC cell proliferation, invasion, and stemness by mediating TRIB3 expression.

### Tan-IIA Suppressed HCC Cell Proliferation, Invasion, and Stemness Through the METTL3/TRIB3 Pathway

**Figure 5.**
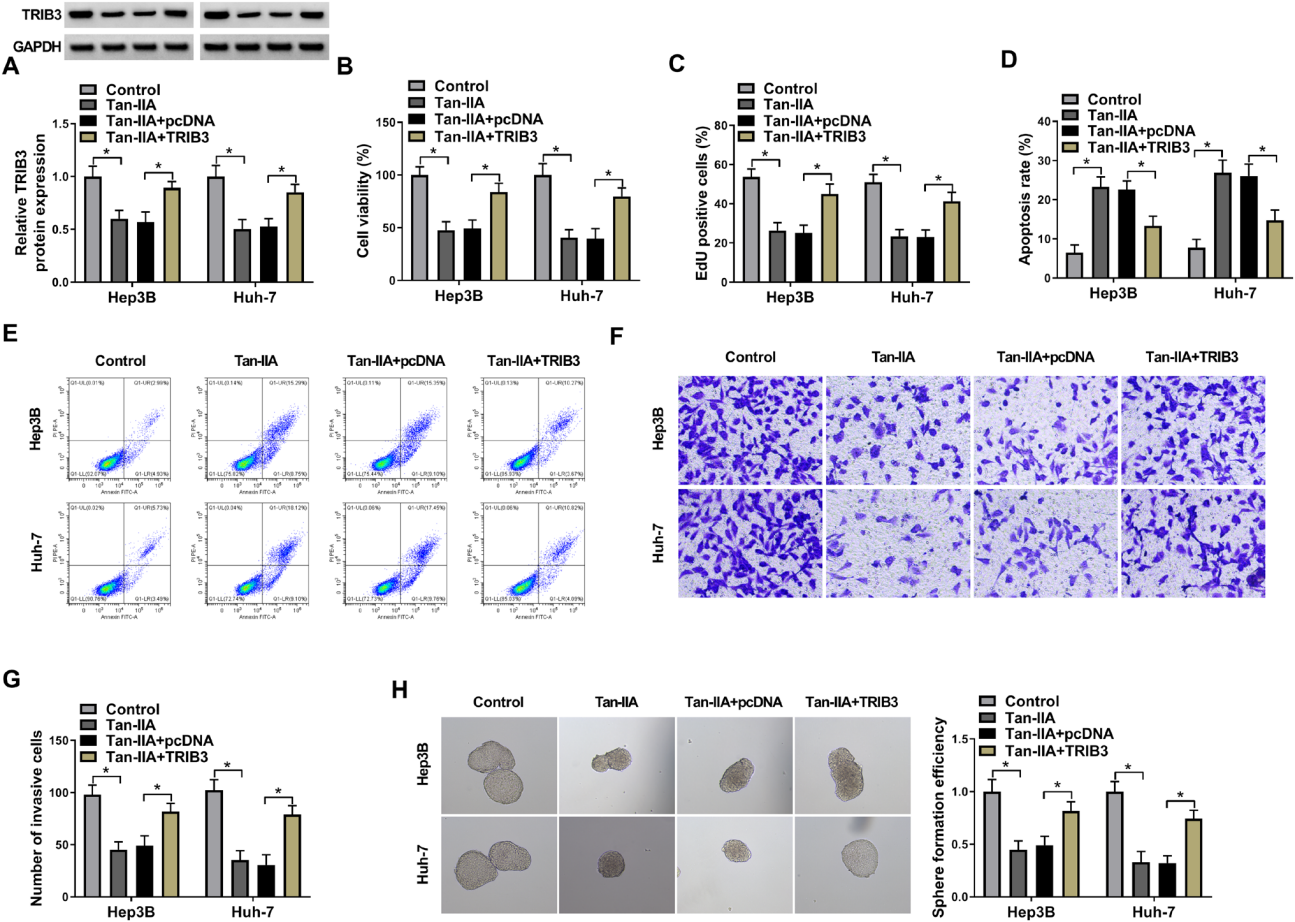
Tan-IIA exerted its function through controlling TRIB3 expression. (A) Relative protein levels of TRIB3 in Tan-IIA-treated HCC cells and Tan-IIA-treated TRIB3-overexpressing HCC cells. (B-H) The viability, proliferation, apoptosis, invasion, and stemness of HCC cells with different treatments. *P < .05 versus control or Tan-IIA+pcDNA.

**Figure 6.**
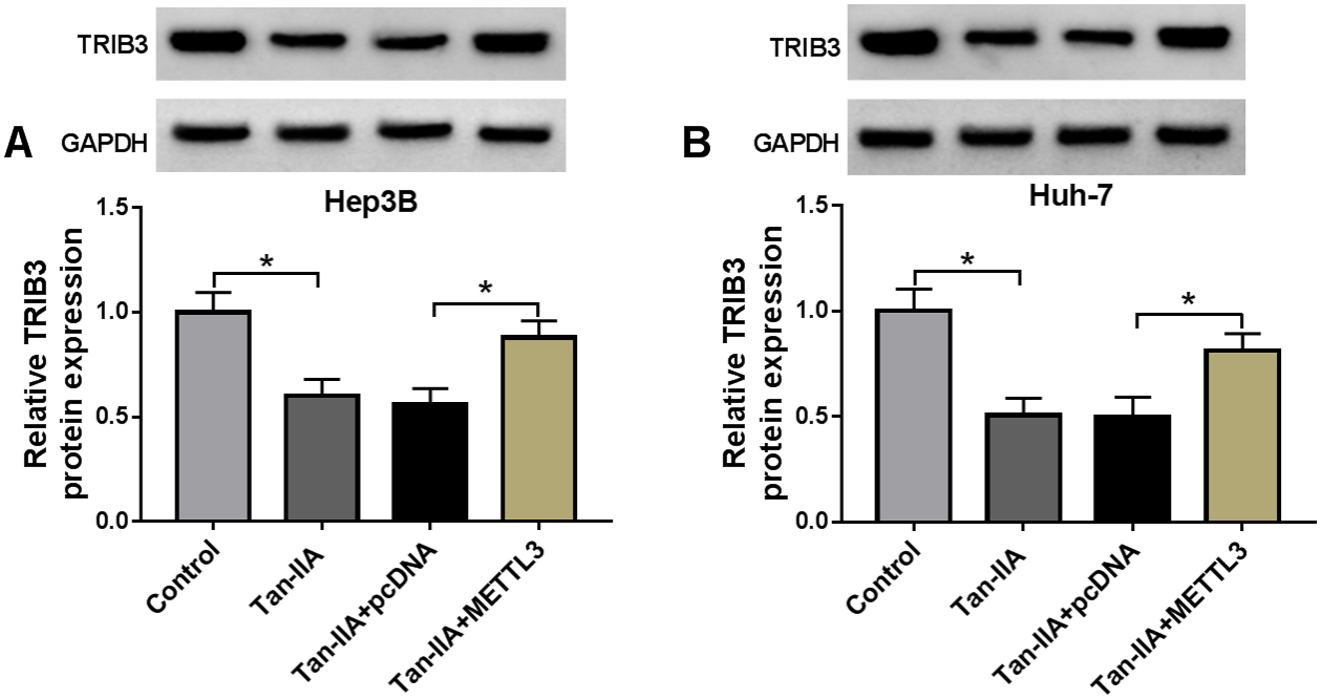
Tan-IIA mediated TRIB3 expression through METTL3. (A and B) Relative protein levels of TRIB3 in Tan-IIA-treated HCC cells and Tan-IIA-treated METTL3-overexpressing HCC cells. *P < .05 versus control or Tan-IIA+pcDNA.

Further experiments were performed to elucidate whether Tan-IIA works by mediating TRIB3 expression. We observed that TRIB3 protein levels were repressed in Tan-IIA-treated HCC cells, whereas the decreased TRIB3 protein levels were overturned after TRIB3 overexpression (Figure 5A). Furthermore, TRIB3 upregulation reversed Tan-IIA treatment-mediated effects on HCC cell viability, proliferation, apoptosis, invasion, and stemness (Figures 5B-H). In addition, Tan-IIA-mediated downregulation of TRIB3 protein levels in HCC cells was reversed after METTL3 overexpression (Figures 6A and B). These outcomes indicated that Tan-IIA mediated TRIB3 expression by METTL3, thereby controlling proliferation, invasion, and stemness of HCC cells.

### Tan-IIA Repressed Subcutaneous Xenograft Growth of Huh-7 Cells

**Figure 7.**
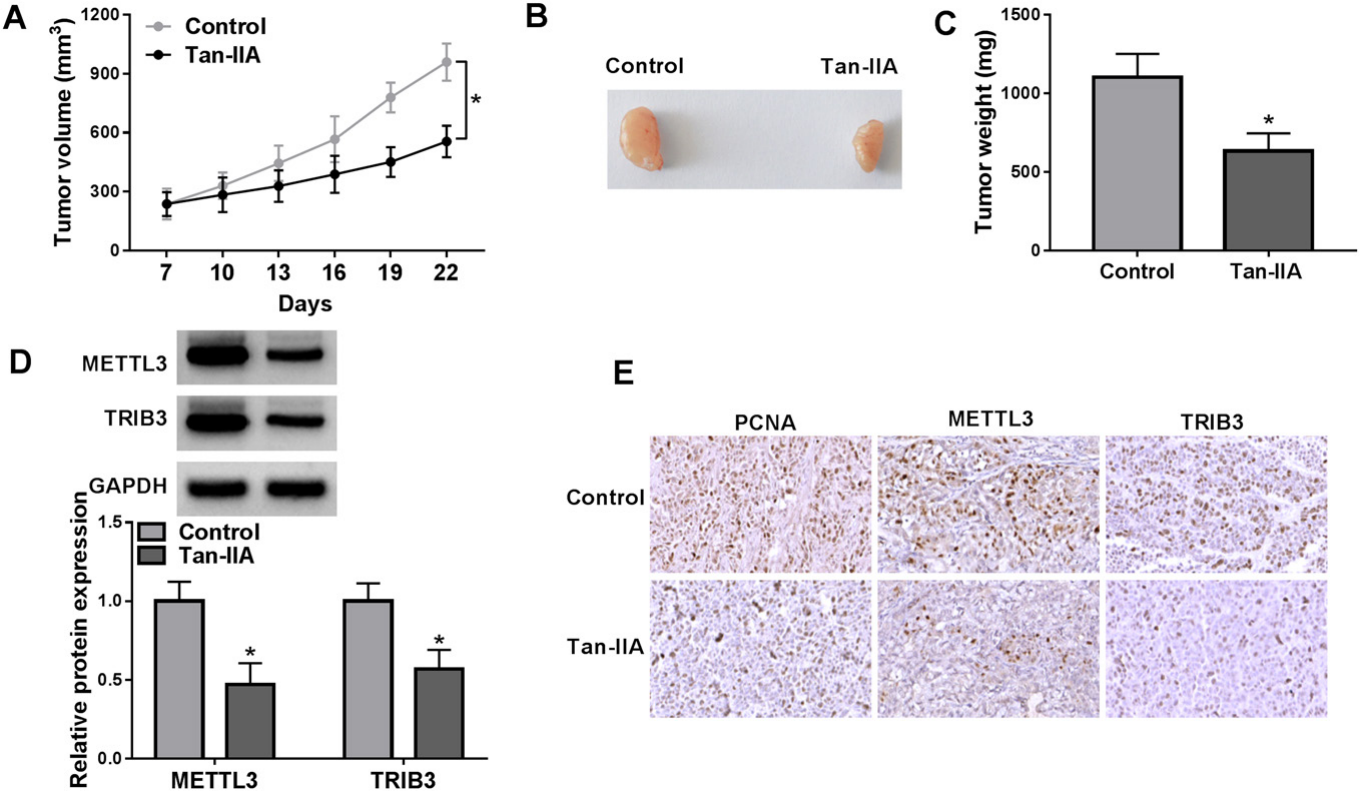
Tan-IIA inhibited the tumorigenesis of Huh-7 cells in xenograft models. (A) Tumor growth curve of subcutaneous xenograft models with or without Tan-IIA treatment. (B) Representative images of excised subcutaneous tumors. (C) Histograms of the average weight of all excised subcutaneous tumors. (D) Relative protein levels of METTL3 and TRIB3 in subcutaneous tumors. (E) Representative images of IHC staining of PCNA, METTL3, and TRIB3 in subcutaneous tumors. *P < .05 versus control.

To verify the role of Tan-IIA in HCC, we established xenograft models using Huh-7 cells. The results displayed that Tan-IIA treatment inhibited tumor growth, both in terms of size and weight (Figures 7A-C). Moreover, the protein levels of METTL3 and TRIB3 were significantly suppressed in Tan-IIA treatment group-derived tumor samples compared to those from the control group (Figure 7D). In addition, the number of PCNA-/METTL3-/TRIB3-positive cells was higher in tumor samples from the Tan-IIA treatment group (Figure 7E). All outcomes suggested an inhibiting influence of Tan-IIA on Huh-7 cell growth *in vivo*.

## Discussion

Multiple genes and multiple factors work together to cause HCC, and one of the mechanisms is the modification of m6A.^23^ Here, we identified a new regulatory mechanism by which Tan-IIA restrained HCC progression by inhibiting MATTL3-mediated TRIB3 mRNA stability.

The regulation of m6A modification is related to a series of physiological and pathobiological behaviors, such as stem cell differentiation, immune regulation, and tumor occurrence and development.^24^ Evidence suggests that aberrant expression of m6A regulators is associated with many aspects of HCC, including the tumor immune microenvironment, drug resistance, metabolic reprogramming, prognosis, and stemness.^25^ As an important methyltransferase, METTL3 exerts a vital role in HCC tumorigenesis.^26,27^ Pan *et al* uncovered that targeting METTL3 can restore the cytotoxicity of CD8^+^ T cells and mediate tumor regression in NAFLD-associated HCC.^21^ Wang* et al* manifested that STM2457, a specific inhibitor of METTL3, can improve the tumor response to lenvatinib in HCC.^28^ Hu *et al* found that METTL3 mediated the m6A modification of SOCS3 mRNA, thus facilitating the stemness and tumorigenicity of liver cancer stem cells.^29^ Importantly, METTL3 is critical in HCC tumorigenesis, but it is unclear whether Tan-IIA works by mediating METTL3. Here, METTL3 was validated to be overexpressed in HCC samples and cells. Moreover, Tan-IIA treatment repressed HCC cell viability, proliferation, invasion, and stemness, accompanied by a decrease in overall m6A levels as well as a downregulation of METTL3 protein levels, implying that Tan-IIA works by repressing METTL3 expression.

In general, METTL3 exerts its role by regulating the m6A modification of RNA.^30^ By bioinformatics analysis and prediction, we found that METTL3 silencing significantly reduced TRIB3 expression in Huh7 cells as well as TRIB3 may bind to METTL3 and contain m6A modification sites. As a pseudokinase, TRIB3 does not have kinase-related activity due to the lack of ATP-binding sites and catalytic residues in its structure, but it can serve as an adapter protein or scaffold protein to regulate cell mitosis, arrest the cell cycle, and interact with various signaling pathways.^31^ Studies have shown that various stress responses can cause TRIB3 to upregulate, such as endoplasmic reticulum stress, oxidative stress, and hypoxia. Moreover, TRIB3 is considered a potential oncogene in solid tumor cells, such as colorectal and breast cancers. Similarly, TRIB3 also exerts a tumor-promoting role in HCC. A previous study manifested that TRIB3 expression is associated with prognosis in postoperative HCC patients, and TRIB3 downregulation decreases the tumorigenesis of HCC cells in animal experiments. The report of Zhu *et al* exposed that Sp2-mediated upregulation of TRIB3 facilitates HCC cell metastasis and invasion. Wang *et al* disclosed that TRIB3 contributes to HCC progression by activating the MAPK signaling. Currently, the m6A modification of TRIB3 mRNA was found in lung adenocarcinoma, but TRIB3 mRNA-associated m6A modification has not been reported in HCC. Here, TRIB3 was overexpressed in HCC samples and cells, and its mRNA levels were positively correlated with METTL3. Moreover, METTL3 enhanced the mRNA stability of TRIB3 by m6A modification, thus promoting HCC cell viability, proliferation, invasion, and stemness. Importantly, Tan-IIA mediated TRIB3 expression through METTL3, thus controlling the proliferation, invasion, and stemness in HCC cells. These results led us to conclude that Tan-IIA inhibits HCC progression by suppressing the METTL3-mediated increase in TRIB3 mRNA stability.

In conclusion, Tan-IIA suppressed METTL3-mediated m6A modification of TRIB3 mRNA, resulting in the repression of HCC cell proliferation, invasion, and stemness, providing a new theoretical basis to support Tan-IIA as a drug against HCC.

## Figures and Tables

**Table 1.** Primers Used for qPCR Analysis

Name		Primers for PCR (5’-3’)
METTL3	Forward	CAGAGGCAGCATTGTCTCCA
	Reverse	ATGGACACAGCATCAGTGGG
TRIB3	Forward	AGATCCCTTTTGATCCGGGC
	Reverse	CCTCCTCTAGGGGTGACGG
GAPDH	Forward	AGAAGGCTGGGGCTCATTTG
	Reverse	AGGGGCCATCCACAGTCTTC

## Data Availability

The analyzed data sets generated during the present study are available from the corresponding author upon reasonable request.
